# Supplementary material for: Relationship Between Brain Insulin Resistance, Carbohydrate Consumption, and Protein Carbonyls, and the Link Between Peripheral Insulin Resistance, Fat Consumption, and Malondialdehyde
Source: Biomedicines. 2025 Feb 7;13(2):404. doi: 10.3390/biomedicines13020404 (PMC11853321; doi:10.3390/biomedicines13020404)
Supplement: Supplementary file 1 [file biomedicines-13-00404-s001.zip › biomedicines-3434637-supplementary.pdf]

Supplementary table S1. Details of de contents of diets.

**Macronutrient**	**Standard diet 5001**	**LFD 12450J**	**HFD 12492i**
**Calories (kcal/g)**	2.86	3.82	5.24
**Carbohydrate (%)**	58	70	20
**Fiber (%)**	5.2	6.5	4.7
**Protein (%)**	28.6	20	20
**Fat (%)**	13.4	10	60
**Saturated fatty acids (%)**	1.4	1.5	9.4
**Ingredients (%)**			
**Sucrose**	3.15	6.9	9.4
**Starch**	21.9	48	0
**Lard**	NS	1.9	31.7
**Soybean oil**	NS	2.4	3.2

NS: not specified
